# A calculation method for optical properties of yolk shell based on deep learning

**DOI:** 10.1371/journal.pone.0302262

**Published:** 2024-05-02

**Authors:** Weiming He, Xiangchao Ma, Jianqi Zhang, Kai Xu, Jingzhou Gao, Shuyao Lei, Changheng Zhan

**Affiliations:** 1 Northwest Institute of Mechanical & Electrical Engineering, Xianyang, Shaanxi, China; 2 School of Optoelectronic Engineering, Xidian University, Xi’an, China; Wayamba University of Sri Lanka, SRI LANKA

## Abstract

The yolk shell is widely used in optoelectronic devices due to its excellent optical properties. Compared to single metal nanostructures, yolk shells have more controllable degrees of freedom, which may make experiments and simulations more complex. Using neural networks can efficiently simplify the computational process of yolk shell. In our work, the relationship between the size and the absorption efficiency of the yolk-shell structure is established using a backpropagation neural network (BPNN), significantly simplifying the calculation process while ensuring accuracy equivalent to discrete dipole scattering (DDSCAT). The absorption efficiency of the yolk shell was comprehensively described through the forward and reverse prediction processes. In forward prediction, the absorption spectrum of yolk shell is obtained through its size parameter. In reverse prediction, the size parameters of yolk shells are predicted through absorption spectra. A comparison with the traditional DDSCAT demonstrated the high precision prediction capability and fast computation of this method, with minimal memory consumption.

## Introduction

The yolk shell structure is a new type of nano multiphase composite material formed by introducing voids between the core and shell through certain technical means on the basis of the core-shell structure. At present, common shell materials include carbon, TiO_2_, and metal shells, and core materials can also be modified according to needs. In this composite structure, the carbon shell can improve conductivity, the TiO_2_ shell increases structural stability, and the metal shell can enhance its absorption capacity. This structure indicates that the yolk shell structure may have excellent optical properties and has been widely studied in fields such as nanophotonics, biosensors, and solar cells [1–5]. The optical properties of the yolk shell is influenced by changes in its structural parameters, including the size of shell, cavity, and core [6, 7]. The freedom of size provides new insights and research freedom for the study and development of novel optoelectronic sensors [8–13]. However, the preparation process of yolk-shell structures is complicated in practical, making it difficult to precisely control their size parameters [14, 15]. Moreover, the use of strong acids and other substances in their fabrication may lead to environmental pollution [16]. Simulating yolk-shell structures using software requires a significant amount of time, computer storage space, and labor costs for subsequent data processing and analysis. Until now, precise control of the optical properties of yolk shell structure remains a challenge [17–20].

**Fig 1:**
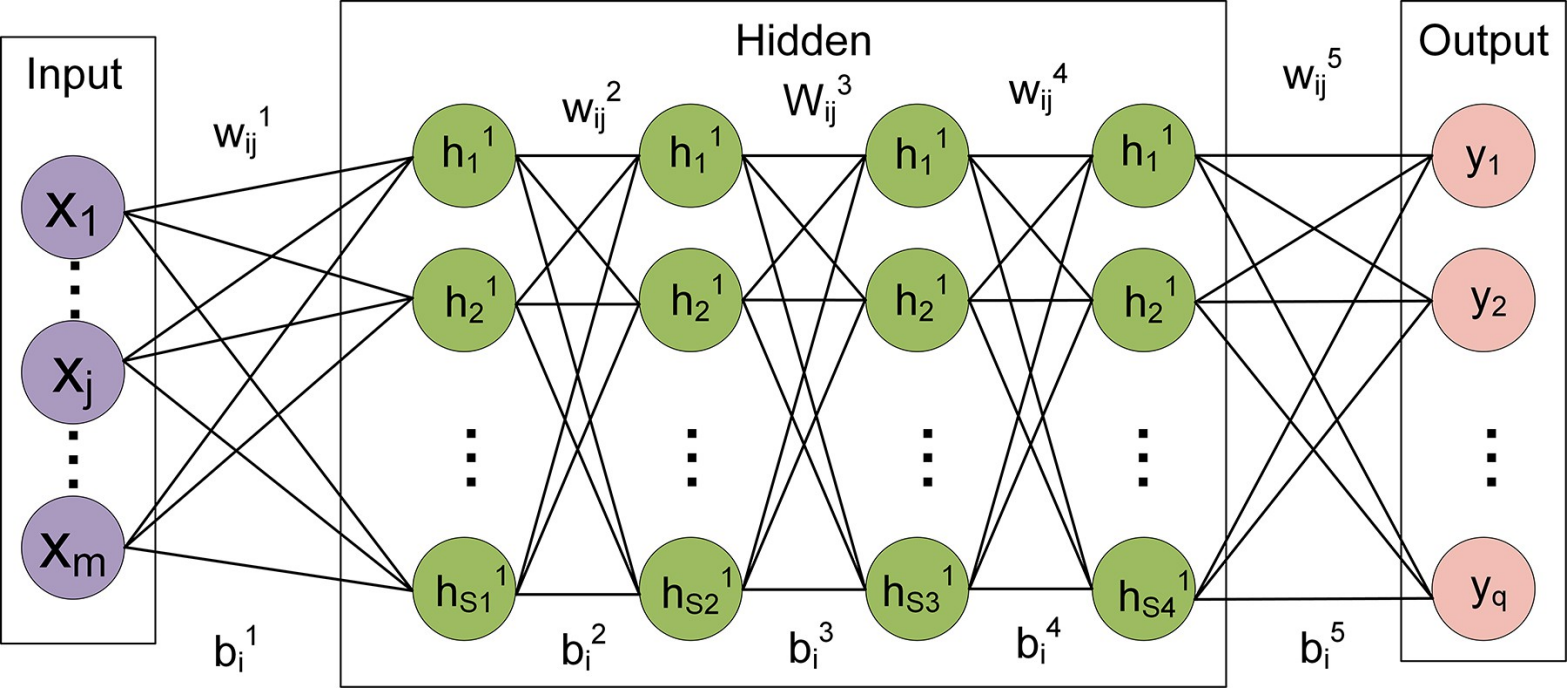
BP basic network structure.

The combination of neural networks with various physical problems for theoretical research is focused in current studies [21, 22]. Since 2006, deep learning has witnessed numerous types of neural networks with diverse functionalities emerging [23–26]. For instance, radial basis function networks address function approximation problems, while Boltzmann machine networks learn probability distributions from raw data for inferring new data [27–30]. In this study, a relatively basic multilayer feedforward neural network called backpropagation neural network (BPNN) was selected to investigate the mapping relationship between the size parameters of yolk shell and their absorption efficiency. The BPNN possesses advantages such as a concise network structure, excellent generalization ability, and strong learning capability, making it one of the widely used neural networks at present [31, 32]. BPNN uses traditional backpropagation algorithm for training, while typical artificial neural network uses gradient descent method. By learning and storing a large amount of input-output data, the BPNN can capture complex nonlinear mapping processes [33]. The BPNN method iteratively reduces network errors through the backpropagation process, and continuously improves network weights and thresholds using the steepest descent method [34, 35]. Compared to the results obtained using the discrete dipole scattering (DDSCAT), the BPNN based equivalent fast calculation method in this paper exhibits significant advantages in terms of computational accuracy, speed, and memory usage. DDSCAT is a numerical simulation program using the discrete dipole approximation (DDA) method, used to simulate the absorption, scattering, and electric field around nanomaterials. As shown in Fig 1, the basic BPNN algorithm comprises two processes: forward propagation of signals and backward propagation of errors. When propagating forward, the input samples are passed in from the input layer, processed layer by layer by each hidden layer, and then transmitted to the output layer. If the actual output of the output layer does not match the expected output (teacher signal), it enters the backpropagation stage of the error.

## Related work

### Ethics Committee approval was obtained from the Institutional Ethics Committee of Northwest Institute of Mechanical & Electrical Engineering to the commencement of the study

**Fig 2:**
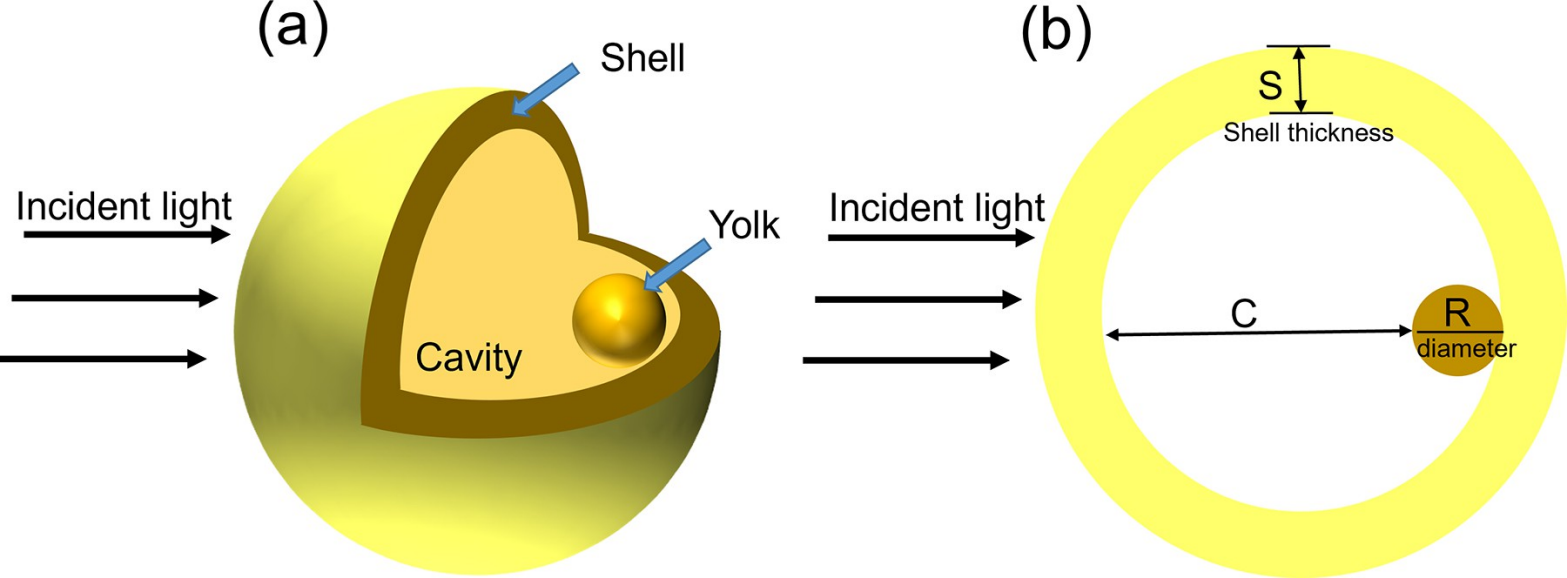
Schematic illustration for geometry of the (a) yolk-shell structure.(b) Front view of yolk shell structure.

The yolk-shell material structure employed in this section consists of three components: a core made of Au, an intermediate air cavity, and an outer shell comprising dielectric material TiO_2_ and metallic material Au. Fig 2(A) illustrates the schematic representation of the yolk-shell structure. As depicted in Fig 2(B), the size parameters of the yolk-shell structure are defined by the core diameter, denoted as R, the distance between the core and the shell, denoted as C, and the shell thickness, denoted as S.

Due to limitations in the DDSCAT software, the dimensions of the structures simulated using discrete dipole approximation require integers. We use DDSCAT to calculate the absorption spectra of approximately 1000 yolk shell structures with different size parameters. The dielectric functions of the shell dipoles were assigned as Au and TiO_2_ to differentiate between the two types of outer shells, yielding a total of 2000 different yolk-shell structures with distinct materials. The absorption spectra of these structures were obtained in the wavelength range of 400–1000 nm, with a sampling interval of 3 nm, resulting in 200 data points. The dielectric function for Au was interpolated from experimental data by Johnson and Christy [36], while the dielectric function for TiO_2_ was interpolated from Devore’s experimental results [37]. The cavity region was set as air with a refractive index of 1 and the yolk shell placed in air.

## Deep learning algorithm

### Algorithm parameter settings

For a given composition and set of size parameters of a yolk shell structure, a unique absorption spectrum can be obtained [38, 39]. Therefore, neural networks are well-suited for constructing this complex mapping relationship, where the size parameters (core diameter, cavity-to-shell distance, and shell thickness) serve as inputs, and the absorption spectrum serves as the output. Once the entire neural network is trained, providing the size parameters of a yolk-shell structure to the network directly yields the corresponding absorption spectrum, significantly improving computational efficiency. In this study, this deep learning-based computational method is referred to as the Back Propagation Neural Network (BPNN) method.

**Fig 3:**
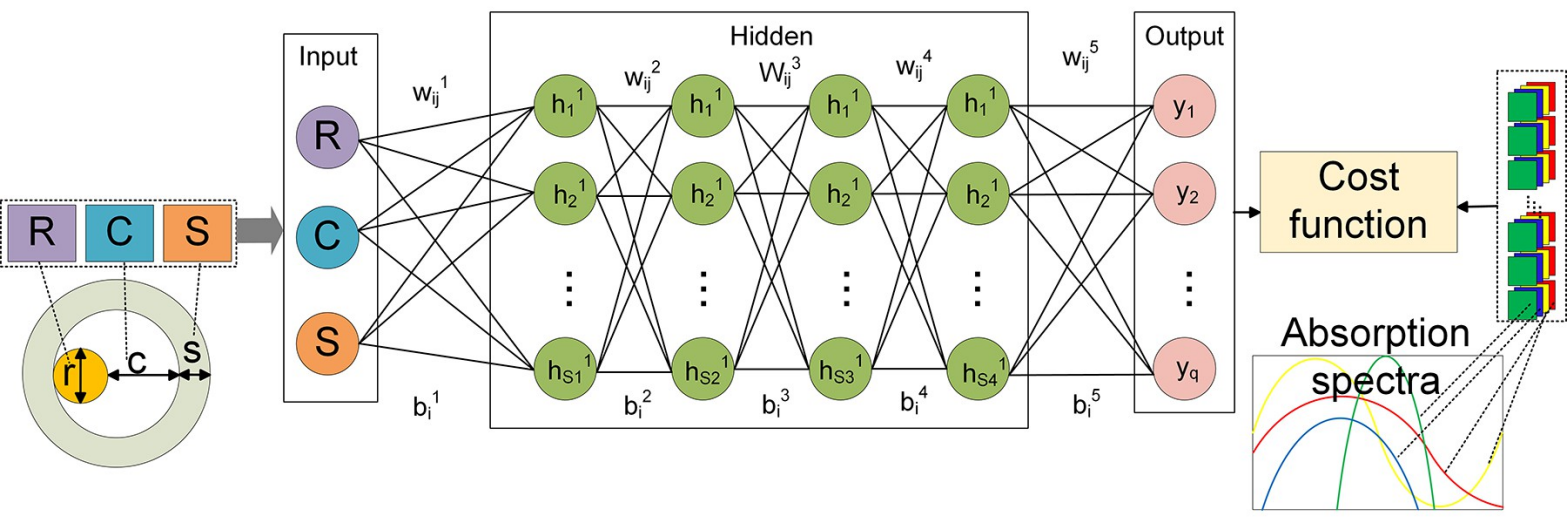
Structure diagram of forward prediction neural network.

In this study, the BPNN will be employed to establish the relationship between the size parameters and absorption spectra. As shown in Fig 3, a multilayer neural network, comprising an input layer, at least one hidden layer, and an output layer, is utilized. Each neuron is responsible for processing the signals from the previous layer and transmitting them to the next layer. To calculate absorption efficiency, BPNN aims to establish a mapping relationship between yolk shell size parameters and absorption spectra [40]. Therefore, the cavity, core, and shell sizes are taken as input parameters, while the absorption coefficient is considered as the output. The training dataset consists of 80% of the simulation results. Each sample consists of 200 data points, each containing three inputs: shell thickness, core diameter, and core to shell distance. These 200 data points are generated in the wavelength range of 400-1000nm with a step size of 3nm. The training dataset is then divided into three parts, with two parts used for parameter selection and model investigation during training. The parameter selection uses grid search method to determine the optimal parameters suitable for the algorithm, the other part is used for model training after the parameters are determined. The remaining part serves as the testing dataset to evaluate the generalization ability of the model. Each subset is used to test the network and optimize the weights, allocating the average component function based on the root mean square error. Finally, the model depth is determined. The optimization of hyperparameters minimizes the offline cost as much as possible. Once the parameters are determined, all data except for the testing dataset will be used for training. The absorption spectra results will be obtained by inputting the size parameters of the yolk-shell. The specific process of constructing the network structure is described as follows.

To establish the mapping between the size parameters of the yolk-shell structure and its absorption efficiency, the network structure depicted in Fig 3 is employed. The three input variables are the core diameter, the maximum distance between the core and the shell, and the shell thickness of the yolk-shell structure, while the output is the absorption spectrum of the yolk-shell structure within the range of 400 to 1000 nm. The above data are all from the calculation of DDSCAT.

The design of hidden layers plays a crucial role in the learning efficiency and generalization capability of deep learning networks. In this study, based on repeated training experiences, a neural network structure with four hidden layers, each containing 1024 nodes, was adopted. The choice of learning rate significantly impacts the training of the neural network. After multiple experimental verifications, an initial learning rate of 0.0001 was selected. If the initial values of the parameters in the neural network are set too large, the output values of the input signals may enter the saturation region of the activation function after being processed by these parameters. Therefore, in this study, the initial values of the weights and thresholds were set to truncated normal distribution random numbers with a mean of 0 and a standard deviation of 1. In the BP neural network, the activation function performs a nonlinear transformation on the input information before passing it to the next layer of the neural network. After multiple validation attempts, the Sigmoid function was chosen as the activation function in this study.


$$f(x)=\frac{1}{1+e^{-x}}$$ (1)

Algorithm optimization is achieved by selecting appropriate parameters, such as the number of hidden layers and activation functions. All of this data comes from grid search, and the training data used is the parameter optimization dataset selected in the dataset. For applications involving optical devices, the maximum energy density of solar radiation received on the Earth’s surface occurs in the visible to near-infrared wavelength range. Therefore, in this study, the wavelength range was selected within the visible to near-infrared spectrum. This choice limits the application range of the BPNN algorithm. When the absorption coefficient wavelength falls within this range, the neural network can obtain absorption spectra with higher precision.

### Forward learning algorithm

A total of 1600 yolk-shell structures with different materials were used for training, while the remaining samples were used as a validation dataset. After training, the testing dataset was employed to evaluate the entire training process. The evaluation primarily focused on two aspects: fitting accuracy and computational efficiency. To validate the fitting accuracy of the BPNN method, the computational results of yolk-shell structures with different shells were compared at various sizes. Additionally, 100 additional data points were generated using the DDSCAT software for result validation purposes.

**Fig 4:**
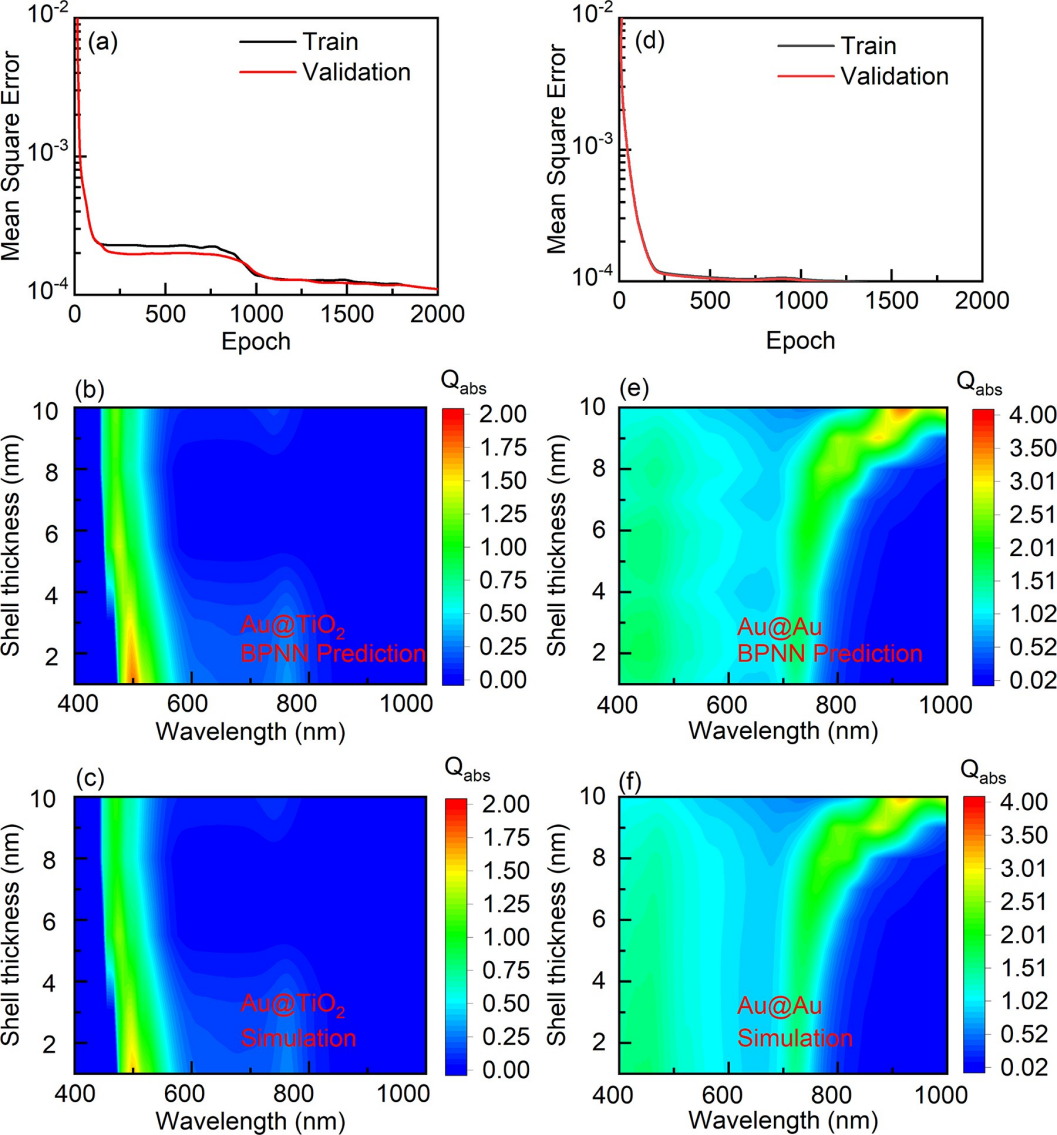
(a) Learning curve of the BPNN model with BPNN algorithm prediction results of Au@TiO_2_, R = 2 nm, C = 4 nm, (b) prediction results, (c) simulation results. (d) Learning curve of the BPNN model with Au@Au, R = 2 nm, C = 4 nm, (e) prediction results, (f) simulation results.

Fig 4 presents a comparison between the predicted absorption spectra and simulated results for yolk-shell structures with TiO_2_ and Au shells when the Au core diameter is 2 nm and the distance between the gold core and the shell is 4 nm. The thickness of the shell is represented on the left axis, with color intensity indicating the level of absorption efficiency. As shown in Fig (4), the simulation results using the BPNN is great agreement with the forward simulations in terms of the absorption efficiency in different wavelength ranges for yolk-shell structures with different materials. The learning curves in Fig 4(A) and 4(D) demonstrate that the BPNN model converges rapidly. As the epochs increase, the loss gradually decreases. The predicted results of the neural network for data not present in the training set closely match the actual results, indicating that the network has converged without overfitting.

In this study, the computational speed of the neural network is another prominent advantage over traditional simulation calculations. Therefore, to further test the model, a set of parameters was used as input to obtain the corresponding absorption spectra using the BPNN algorithm. If the calculations were performed using the DDSCAT software, it would take approximately 5 hours from the initiation of the design model to the convergence of a set of parameters. In contrast, using the proposed forward prediction network in this chapter, the computation time is only 3.9 ms. For the training time, when the parameters are determined and the training data is ready, the training of the neural network will also be completed within 30 minutes, which greatly improving efficiency.

The above results demonstrate that the proposed algorithm in this section not only achieves the same accuracy in obtaining absorption spectra as the DDSCAT algorithm but also allows for more efficient calculations, particularly for models that consume a significant amount of time in DDSCAT calculations. The BPNN algorithm offers a size accuracy range that far exceeds that of the DDSCAT algorithm. Therefore, in this section, the BPNN algorithm was also utilized to generate yolk-shell structure sizes with lower resolutions. By expanding the size parameters, further investigation into the impact of size parameters on the absorption efficiency of yolk-shell structures can be conducted with greater precision.

**Fig 5:**
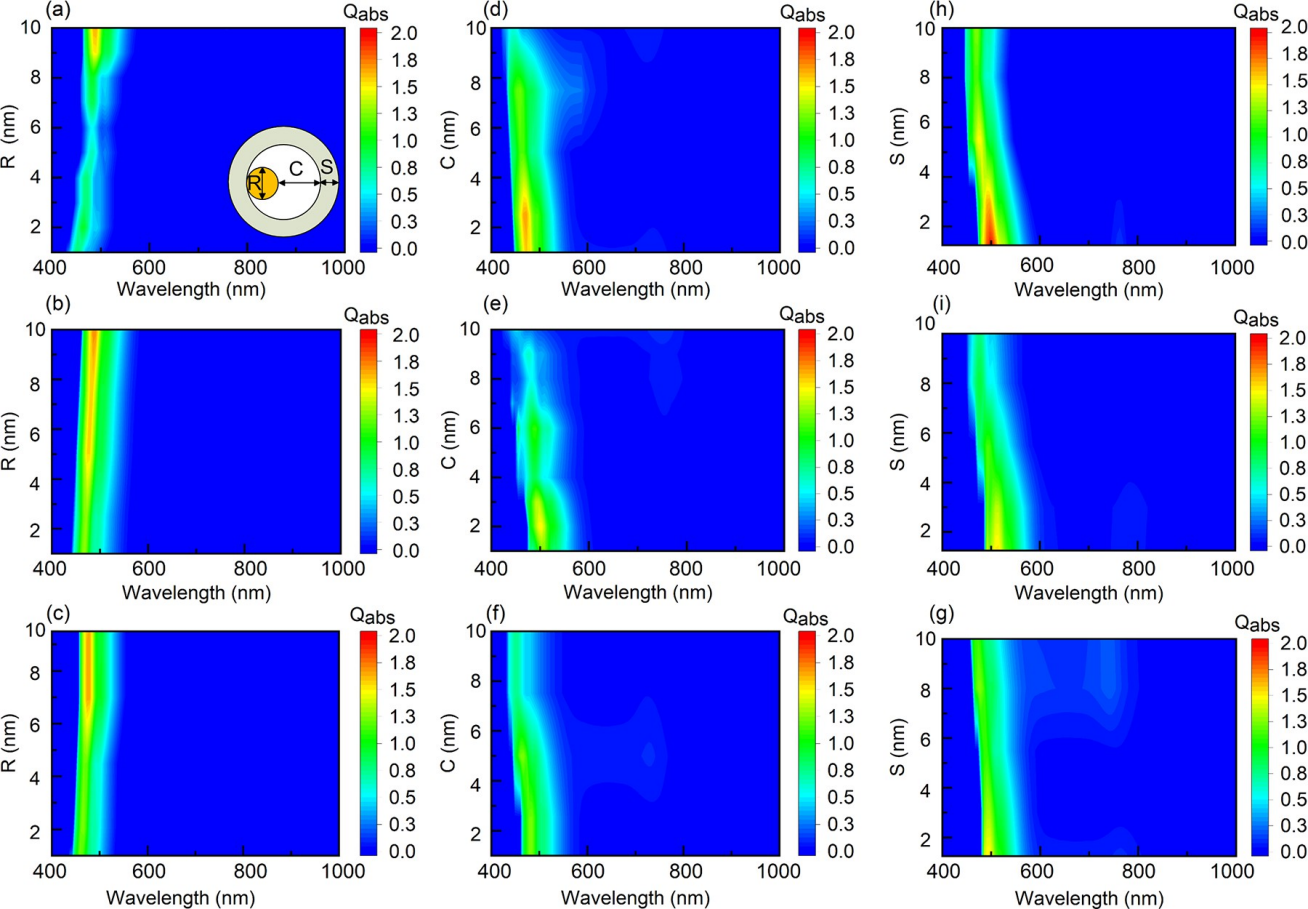
BPNN prediction of Au@TiO_2_. An absorption spectrum with a distance of 5 nm from the Au core to the shell, (a) S = 4 nm, (b) S = 7 nm, and (c) S = 9 nm; The diameter of Au nucleus is 6 nm, (d) S = 3 nm, (e) S = 5 nm, (f) S = 7 nm; The diameter of the Au nucleus is 7 nm, with (g) C = 6 nm, (h) C = 7 nm, and (i) C = 9 nm.

Fig 5 illustrates the dependency of yolk shell structures on different size parameters within the 400 to 1000 nm wavelength range. For each plot, size parameters that are not labeled in the figure remain constant during data generation. The left axis represents the parameter that varies solely within the plot, while the varying absorption coefficients are represented by increasingly darker colors on the right side. In Fig 5, the size parameters of the yolk shell structures were expanded using the BPNN method. Each result in every plot consists of 90 data sets, with a resolution of 0.1 nm between the variable sizes represented on the right axis.

Fig 5(A)–5(C) depict the trend of absorption efficiency of yolk shell structures with varying gold core diameters. It can be observed that as the gold core diameter increases, the absorption intensity gradually rises, and there is a noticeable redshift in the absorption peak position. Additionally, with increasing diameter, the responsive range of the absorption spectrum widens. When the gold core size remains constant, for structures with shell thicknesses of 4 nm, 5 nm, and 7 nm, it is apparent that with smaller cavities exhibit higher absorption intensity, while the width of the absorption spectrum remains relatively stable. Fig 5(D)–5(F) present the variation of absorption efficiency of yolk-shell structures with different core-to-shell distances. It can be observed that yolk-shell structures with smaller cavities have higher absorption efficiency.The peak of absorption efficiency is redshift with the increased of the cavity size. Fig 5(G)–5(I) demonstrate the impact of shell thickness on the absorption efficiency of yolk shell structures. It can be observed that increasing the shell thickness significantly shields the yolk shell structure, leading to a significant decrease in absorption efficiency. Moreover, the responsive range of the absorption spectrum narrows with increasing shell thickness. Within the study range, yolk shell structures with dielectric shells primarily exhibit absorption spectra concentrated in the visible light range. The absorption intensity is more influenced by size parameters compared to the absorption peak position. Therefore, for yolk shell structures with TiO_2_ shells, those with medium cavity distances, larger gold cores, and thinner shells exhibit better absorption intensity.

**Fig 6:**
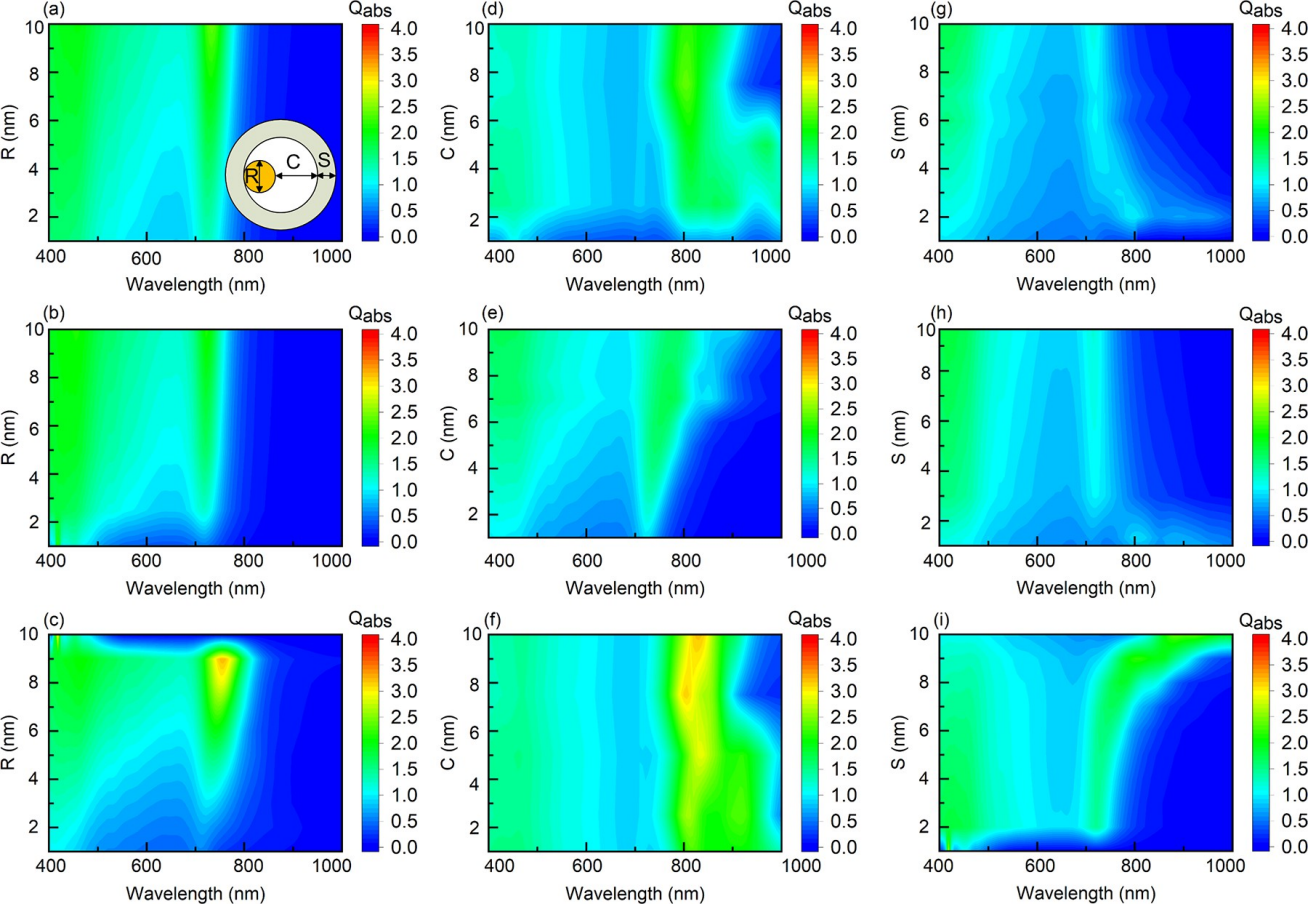
BPNN prediction of Au@Au. The absorption spectrum of Au with a distance from the core to the shell of 5 nm, (a) S = 4 nm, (b) S = 7 nm, (c) S = 9 nm; The diameter of Au nucleus is 6 nm, (d) S = 3 nm, (e) S = 5 nm, (f) S = 7 nm; The diameter of the Au nucleus is 7 nm, (g) C = 5 nm, (h) C = 7 nm, and (i) C = 9 nm.

For the Au@Au yolk shell structure, different trends can be observed from the results. Fig 6 presents the absorption efficiency results of Au@Au for varying parameters. It can be seen that the absorption efficiency of Au@Au is significantly greater than that of Au@TiO_2_ in terms of absorption intensity. Notably, larger-volume Au@Au structures exhibit two absorption peaks, and the responsive width of these peaks is also larger. This suggests that Au@Au structures may possess a wider absorbable wavelength range and higher absorption efficiency.

The trained neural network can better expand its output to obtain absorption coefficients for more combinations of parameters. The BPNN algorithm used here requires significantly less resources for computational methods that produce equally dense output data comparable to DDSCAT. Comparing the two computational methods for a specific combination of geometric parameters, running the DDSCAT software on a computer with an i7 chip would take at least 2 hours to complete the convergence and output of absorption efficiency. And the neural network model used in this study can output the absorption spectra for a given set of parameters in less than a second after the network has been trained. On the other hand, for sampling density in this study with same output of neural netwaork, would consume an astonishing amount of time, taking about 5 years to complete, during which the computer must run continuously without being turned off. Therefore, in this research, using the neural network approach is far superior in terms of computational speed and lower energy consumption compared to traditional software.

### Reverse prediction algorithm

After achieving gerat accuracy in forward design, the reverse path design needs to be further explored. For devices based on yolk-shell nanostructures, the reverse parameter design of the yolk-shell nanostructure is often required after determining the operating environment and the corresponding absorption peak position or intensity. Therefore, it is necessary to design and investigate algorithms that are different from forward prediction. The training data used in the reverse prediction algorithm is sourced from DDSCAT. Specifically, the absorption spectra of yolk shell nanostructures are used as input parameters to output the size parameters of the yolk shell structure, including the gold core diameter, denoted as R, the maximum distance between the gold core and the shell denoted as C, and the shell thickness denoted as S. However, compared to forward prediction algorithms, the reverse design path is more complex and more sensitive. This is because in this study, there are 200 data points for the absorption spectra, while there are only 3 data points for the size parameters, resulting in a significant imbalance between the input and output dimensions.

In this study, a neural network structure was initially designed using the BPNN network, with the absorption spectra as input and the size parameters of the yolk-shell structure as output. The design of this reverse prediction network is the same as that of the forward prediction network. However, upon testing, the output results of this reverse model were not satisfactory. The specific reasons are as follows: (1) Although the design of the model can continue to use the previous sampling results, the mismatched input-output relationship (input of 200 data points and output of 3 data points) may lead to information loss, especially in complex structures; (2) The neural network structure is not sufficiently deep to handle complex nonlinear tasks, and excessively deep connected layers can cause gradient vanishing, thereby increasing the complexity of the model.

**Fig 7:**
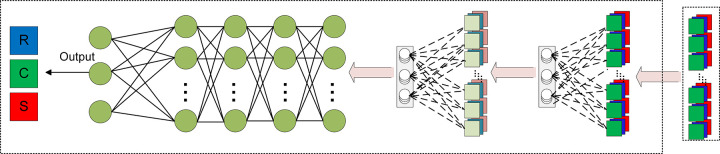
BPNN reverse design architecture.

In order to utilize the absorption spectra as input and achieve more accurate retrieval of geometric parameters in the output, adjustments were made to the forward prediction network. The spectrum data was first connected to two typical convolutional neural networks (CNNs), with the first CNN comprising layers with 4x3x1 kernels and the second CNN with layers containing 1x2x3 kernels. These CNNs were then connected to a neural network with four hidden layers, as shown in Fig 7. By performing computations through the first two convolutional layers, the correlation between the input and output data was effectively enhanced. Running this reverse prediction network allows for the prediction of geometric parameters by providing the absorption spectrum, and the results closely resemble the parameters generated by the forward prediction of absorption efficiency spectra.

From the results in this work, the size parameters of these yolk-shell structures are interdependent and highly sensitive to variations in the absorption efficiency. Therefore, for the validation of reverse prediction, the method involves inputting the absorption spectrum and obtaining the corresponding size parameters as output. These output size parameters are then fed back into the forward prediction network to plot the predicted absorption spectra against the true size parameters and examine the relationship between the predicted size parameters and the true absorption spectra.

**Fig 8:**
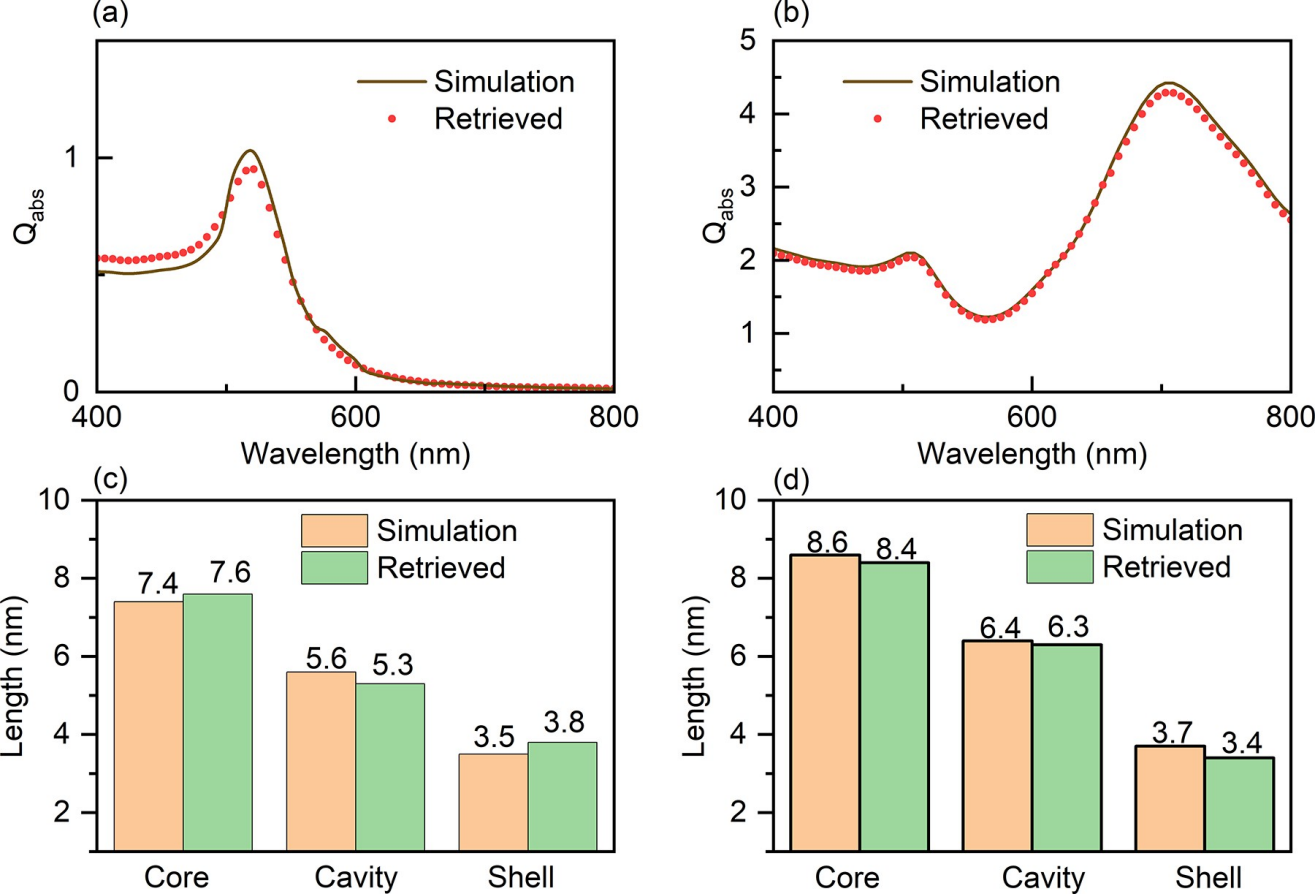
Reverse prediction results. (a) Au@TiO_2_ Predicted results and actual data, (b) Au@Au Comparison of size parameters between predicted results and real data, reverse prediction and forward prediction, (c) Au@TiO_2_, (d) Au@Au.

The comparison between the reverse prediction network and the true results is shown in Fig 8. From the figure, it can be observed that the neural network’s configuration is essentially correct by comparing the simulated spectra with the corresponding predicted spectra. Fig 8(B) and 8(D) represent the spectra of Au@Au yolk shell structures under the same conditions. Although there are slight differences between the simulated results and the machine learning predicted spectra, the absorption peak positions and intensities of the absorption spectra are essentially consistent. Furthermore, Fig 8 also demonstrates that good reverse prediction results can be obtained for yolk shell structures with different material shells. This indicates that the proposed prediction model in this section is applicable to different materials.

### Conclusion

A novel learning network based on the BPNN algorithm is proposed in this paper for predicting the absorption spectra of yolk-shell structures with metal cores. The results demonstrate that the prediction method proposed in this study not only achieves the same level of accuracy as the DDSCAT method but also significantly improves computational efficiency. By utilizing the BPNN approach, new insights are provided for modeling yolk-shell composite structures of different sizes, making it an effective computational tool for predicting absorption spectra in metallic composite structures.

## Supporting information

S1 File Training dataset and prediction results. (XLSX)
